# Coordination between primary and secondary care: the role of electronic messages and economic incentives

**DOI:** 10.1186/s12913-017-2096-4

**Published:** 2017-02-17

**Authors:** Antonella La Rocca, Thomas Hoholm

**Affiliations:** 1 Akershus University Hospital, Sykehusveien 25, Lørenskog, 1478 Norway; 2 Department of Innovation and Economic Organization, BI Norwegian Business School, Nydalsveien 37, Oslo, 0484 Norway

**Keywords:** Health care, Coordination, Communication, Policy, Integrated care, Health care network

## Abstract

**Background:**

In Norway, a government reform has recently been introduced to enhance coordination between primary and secondary care. This paper examines the effects of two newly introduced measures to improve the coordination: an ICT-based communication tool/standard and an economic incentive scheme.

**Method:**

This qualitative study is based primarily on 27 open-ended interviews. We interviewed nine employees at a hospital (the focal actor), 17 employees from seven different municipalities, and a representative of a Regional Health Authority.

**Results:**

ICT-based communication is perceived to facilitate information exchange between primary and secondary care, thus positively affecting coordination. However, the economic incentive scheme appears to have the opposite effect by creating tensions between the two organizations and accentuating power asymmetry in favor of secondary care.

**Conclusions:**

The inter-organizational nature of coordination in health care makes it crucial for policymakers and management of care organizations to conceive incentives and instruments that work jointly across organizations rather than at only one of the health care organizations involved. Such an approach is likely to favor a more symmetrical pattern of collaboration between primary and secondary care.

## Background

Health care provision is considered a collective work, but health care organizations tend to work autonomously [1]. The tendency of health care organizations to work in ‘distinct silos’ [2] can be associated with different issues. It is related to existence of strong professional boundaries [3], which inhibit collaboration and knowledge sharing across boundaries [4]. As part of a care continuum, professionals in the two settings might compete for jurisdiction over certain tasks [5]. Jurisdiction, defined as the “the link between a profession and its work” [6], is crucial for professionals because it is their means of continued livelihood [7]. The unequal distribution of power among health care organizations is an obstacle to collaboration. Hospitals, providing most health care services and ultimately receiving most of the resources, traditionally enjoy a privileged position [8]. Primary care organizations and those working within them depend on hospitals for many aspects of care provision; they are typically given much less resources to provide follow-up or preventive care in the home or community setting that primary care organizations deliver [8]. The evidence suggests that improved coordination among health care contributes to enhance quality of care and efficiency performance [9]. Consequently, policymakers are increasingly tinkering with mechanisms to integrate activities across health care organizations [10–13]. New models of organizing and delivering care have been developed in the pursuit of transcending primary and specialty care boundaries and improving care coordination [14]. However, it has been observed that mechanisms through which coordination is to be achieved are not well understood [15] and “rarely identified in relevant policies” [16].

Coordination here refers to “the deliberate organization of patient care activities between two or more participants involved in a patient’s care to facilitate the appropriate delivery of health care services” [17]. In this study, we will focus on care coordination between primary and secondary care. A timely exchange of appropriate information among health care professionals has been identified as a prerequisite for care coordination [17]. There are, in particular, two situations/processes in which communication between hospital professionals and professionals at the primary level of care is critical: When the patient is transferred from municipal care to the hospital (admission or readmission), and when the patient is transferred from hospital to municipal nursing care (discharge). A deficit in communication when patients are transferred across health care providers can cause ineffective care for patients [18]. Information exchange during the hospital discharge process is fundamental for decision-making (preceded by the assessment of patient’s conditions) of the after-hospitalization care service patients need [19]. Inappropriate or delayed information during discharge process can result in patients staying at hospital longer than necessary, waiting for the appropriate follow up care service to be arranged, or receiving inappropriate follow up care services, which might lead to increase in readmissions [20]. According to Peikes et al. [21], intervention to improve care coordination should put more emphasis on preventing readmissions, which are the costlier health care events.

Supporting communication among health care organizations has been identified as way to improve organizational efficiency and effectiveness as well as offering the opportunity to improve patient care [22, 23]. Since the 1990s, information and communication technologies (ICT) have attracted increasing attention as facilitating tools in the health care sector. Health information technology has been seen to have “the potential to improve coordination by making information electronically available” [24]. At a hospital, managers consider “IT as the key tool for achieving a better information flow and better services, as well as for complying with organizational objectives regarding high quality in patient care treatment” [25]. At primary care, the implementation of ICT systems has been shown to be a catalyst in establishing new communication procedures [26]. However, tensions generated by ICT system, in particular by the introduction and use of electronic health records (EHR), have been also evidenced in a number of studies [27–30]. One recurrent concern here is the tension between structure and flexible documentation [30]. It has been found “that physicians who predominantly dictated their notes appeared to have worse quality of care, especially as compared to physicians who used structured EHR documentation” [28]. Bossen [27] emphasizes that there are different “representations at work in IT technology” that “enable cooperation, coordination, accountability and control,” and that these have to be “balanced off against each other.”

A more accurate and comprehensive sharing of information through ICT systems is not the only area of intervention to enhance care coordination. Improvement of care coordination has been associated with several financial strategies, including both incentives and penalties [31]. Activity-based funding (ABF) systems, prospective reimbursement based on patient case mix, have been generally considered successful in improving financial transparency and creating incentives for technical efficiency [32]. However, it has been observed that they have offered little to create incentives outside of the hospital, in addressing the lack of coordination across health care providers and settings [33]. As result, there is increasing interest in exploring financial incentives for care coordination across providers and settings [33]. The key question to be addressed is: which incentives should be operated at the interface between primary and secondary health care in order to motivate professionals to use their resources to achieve the best possible health outcomes? [34, 35]. Accountable care organizations (ACOs) and bundled payments are designed to create monetary incentives for coordinated care. These measures range from “penalizing hospitals with higher-than-expected readmission rates, to rewarding primary care providers when patients receive higher-value care, to providing incentives for the adoption of electronic health records” [36]. The ambition with this incentives scheme is that coordination will “improve value by ensuring that the right care is provided in the right place at the right time” [36].

In this study, we examine the case of Norway, as the Norwegian government, on January 1, 2012, has introduced a coordination reform to enhance care coordination and more efficient use of resources in the Norwegian health care system [37] designing instruments along the aforementioned lines. The government introduced a new ICT solution to facilitate communication between professionals at the primary and secondary levels and opted for an incentive scheme that aims to stimulate a change in the way primary care manages the in/out hospital transition of patients. In Norway, health care is publicly funded, primary care is managed by 429 municipalities, while specialist health care is managed by four state-owned Regional Health organizations/authorities. Key objectives of the reform have been: 1) more patients should be taken care of in primary health care instead of specialized care; and 2) discharge from hospitals should take place earlier [38]. To pursue these objectives, the Norwegian Ministry of Health and Care Services has set as priority to improve communication and coordination between hospitals and municipal health care systems [39]. The Norwegian government opted for a renewal of the information and communication system with the introduction of electronic health care messages (PLO, an acronym for ‘Pleie- og omsorgsmeldinger’). The effort put in the development of an electronic messaging system to better connect primary and secondary care is also due to a regulation in Norway that clearly indicate responsibilities, administrative and financial structures of the two systems of care. This regulation prescribes that it is a unit (‘ordering office’ or ‘purchasing unit’) at the primary level of care, which has the responsibility and authority to assess individual needs, formulate contracts, decide and order care services and control outcomes of care the providers deliver [40].

Besides the ICT-based communication, the government moved some care funding (5 billion Nok) from hospitals to municipalities, which, in turn, became responsible for co-financing hospital admissions of patients in need of selected somatic health care services. This measure’s aim is twofold: reduce hospital costs by reducing unnecessary admissions and providing more resources to primary care thereby trying to revise the ‘privileged position’ of hospitals [8] in favour of a potential empowerment of primary care. Furthermore, according to a payment regulation reform (the so-called *Betalingsforskrift*), when a municipality is unable to receive a patient declared ready for hospital discharge, it must pay a fee (4,500 Nok) to the hospital. This fee is meant to compensate the hospital for every 24 h the patient has to stay in the hospital. This measure aims to shorten length of stay after the scheduled discharge date, thus facilitating patient transition and reducing waiting time for hospital treatment [41]. Overall, these measures fit into the broader, and common across countries, agenda of improving cost-efficiency of hospitals and the consequent effort to strengthen primary health care [42, 43].

Previous studies have warned about reforms not being ‘neutral’ instruments and not always producing intended effects [44]. This makes it interesting and relevant to examine the effects of the measures promoted by the coordination reform to improve care coordination between a hospital and some municipalities in the relevant catchment area. In this qualitative paper, we describe how the two instruments – electronic communication and a new economic incentive system – settled by the government in pursuit of better coordination between primary and secondary care, have been translated into practice by health care professionals belonging to different health care settings. We aim to explore how the two instruments in place might interact with each other and/or and which effects these measures might produce independently of the declared aim ascribed to them. Our study adds to existing knowledge about care coordination and the mechanisms to enhance it [2, 15, 16, 23, 45, 46]. More narrowly, this study engages into an ongoing debate in the literature about the attempts to reform health care in Norway. This research can be seen as a follow-up of previous studies, which have explored care coordination mechanisms conducted before the coordination reform was introduced [19] and have delineated possible negative side effects of the reform [38]. It also complements a study exploring the effects of the reform, thus highlighting an increased need for inter-municipal cooperation after the reform was introduced [47].

## Method

This study is part of a larger longitudinal research project investigating changes within and across health care organizations when introducing electronic messaging and mobile solutions. The study has been approved by the Data Protection Official for Research (ref. no.14-022) and cleared with the “REC South East”, a Regional Committees for Medical and Health Research Ethics (REK) in Norway. In our study we have only gathered data from and about healthcare professionals. All informants were asked to participate in our qualitative research project, and they got written materials regarding the design and aims of the research project in due time before the interviews. At the start of each interview, informed consent was confirmed by participants and anonymity was guaranteed.

### Recruitment of participants and data collection

The primary material in this paper derives from 27 face-to-face, semi structured interviews conducted with key informants between December 2013 and January 2015 (see Table 1) in the health care sector at the secondary level of care (one focal hospital that, for confidentiality reasons, will be “the Hospital” hereinafter) and at the primary level of care (seven related municipalities whose names have been anonymized). For selecting respondents, we used a convenience sampling asking for assistance from the Department A of the Hospital. Based on its knowledge about our project and on past collaboration with hospital personnel, the Department A provided us a list of potential respondents belonging to different divisions and covering different roles. We subsequently contacted the potential respondents by e-mail, providing information about the project and checking their availability for face-to-face interviews. We followed a similar procedure for selecting the respondents at the municipality level. Based on an initial contact list provided by Department A, we contacted by e-mail the municipalities in the catchment area of the Hospital explaining the objective of the study and asking availability for an interview. Snowball sampling was also used to recruit more informed participants within the contacted municipality. Finally, one of the respondents (R27) was purposefully selected because of the respondent’s role as representative of the Regional Health Authority.

**Table 1:** List of Respondents

Respondent	Organization	Department/Ward
R1	Hospital	Department A
R2	Hospital	Department A
R3	Hospital	Ward B
R4	Hospital	Ward C
R5	Hospital	Ward C
R6	Hospital	Ward D
R7	Hospital	Department B
R8	Hospital	Department A
R9	Hospital	Ward E
R10	Mal Municipality	IT-service unit
R11	Mal Municipality	IT- service unit
R12	Mal Municipality	IT- service unit
R13	Mal Municipality	Home care unit
R14	Mal Municipality	Home care unit
R15	Mal Municipality	Ordering Office
R16	Mal Municipality	Ordering Office
R17	Mel Municipality	Health and Care Services
R18	Mel Municipality	Health and Care Services
R19	Mil Municipality	IT-service unit
R20	Mil Municipality	Ordering Office
R21	Mil Municipality	Health and Care Services
R22	Mol Municipality	Health and Care Services
R23	Mol Municipality	Health and Care Services
R24	Mul Municipality	Service Management Unit
R25	Myl Municipality	Ordering Office
R26	Mes Municipality	Home care unit
R27	Regional Health Authority	Representative

At the primary care level, we interviewed 17 care professionals working in eight different municipalities, mostly at the ordering office, which is the unit that has the authority to decide what care services a patient will need/receive when discharged from a hospital. At the Hospital, we interviewed clinicians of four divisions and five respondents with no strictly medical responsibility. We also interviewed a representative of the Regional Health Authorities. Secondary data in the form of official reports to the health authority, official hospital and municipalities’ internal documents and records, press releases, and previously published articles on this matter were also collected to achieve the study’s objectives. The multiple data sources enabled cross checking through triangulation, and the prolonged engagement of the authors within the field allowed for a thorough appreciation of the context [48].

In terms of how the interview guide was devised, we have been guided by our initial frame of reference [49] by focusing on the link between the two measures in place and improvement of care coordination. At the beginning of the interviews, all the participants were asked the question: ‘*Could you describe your role, tasks and responsibilities*?’ This question was broadly formulated for capturing eventual and spontaneous aspects of the collaboration between the respondent and the counterpart organization not likely to emerge with more specific questions. The second open-ended question allowed respondents to elaborate on particular areas that they regarded as important: ‘*What are the main issues of concern in collaborating and coordinating with your counterpart*?’ Through questions three: ‘*Do you see any benefit of the new communication system for your work practices and for coordination between primary and secondary care*?’ and four ‘*Do you see any challenges of the new communication system for your work practices and for coordination between primary and secondary care*?’ we aimed to explore the actual experience of respondents with using the new ICT tool, both for their internal documenting practices and for communicating and coordinating across organizational boundaries. The fifth question, ‘*What are the effects of the new incentive system (co-financing and payment regulation) for your work practices and for coordination between primary and secondary care*?’ was left for the ending because it was supposed to be the most sensitive due to the association between quality of care and economic issues. All the interviews lasted from 45 to 60 min. We have recorded and transcribed 21 of them. For the other six (when respondents declared not feeling comfortable with speaking in front of a recorder or when, for technical reasons, we have not been able to record the interview), we took detailed notes during the interviews, including quotes from the speech, and we merged notes just after the interview to build an accurate and detailed report. This should have partially mitigated the lack of direct recording.

### Data analysis

Interview transcripts and detailed notes were manually coded following a grounded theory approach to extract themes from the interviews [50]. The two authors read each transcript and notes and listed significant statements. Next, the authors reviewed the transcripts and notes for meaning and labeled the participants’ statements [51]. The authors then decided jointly what they regarded as a significant statement. The joint decision was made to achieve consensus about the manner in which to organize the statements into distinct themes [52]. This allowed us to enrich our analysis with multiple interpretations and to achieve subjective understanding across coders [53, 54]. This method generated varied themes and sub-thematic areas that captured the essence of the debate. Inspired by the logic of second-order analysis [55], we have tried to offer (in the discussion section) an “interpretation of what transpired that goes beyond that offered by the informants” [56]. This approach is in line with ‘systematic combining’ – an ‘abductive approach’ to research, which has been described as a nonlinear, path-dependent process of combining efforts to match theory and reality [57]. Trustworthiness of data was thus enhanced through searching for alternative explanations and linking the findings and conclusions to data and evidence from the literature [48].

## Results

### The uses of the new ICT solution

**Fig. 1:**
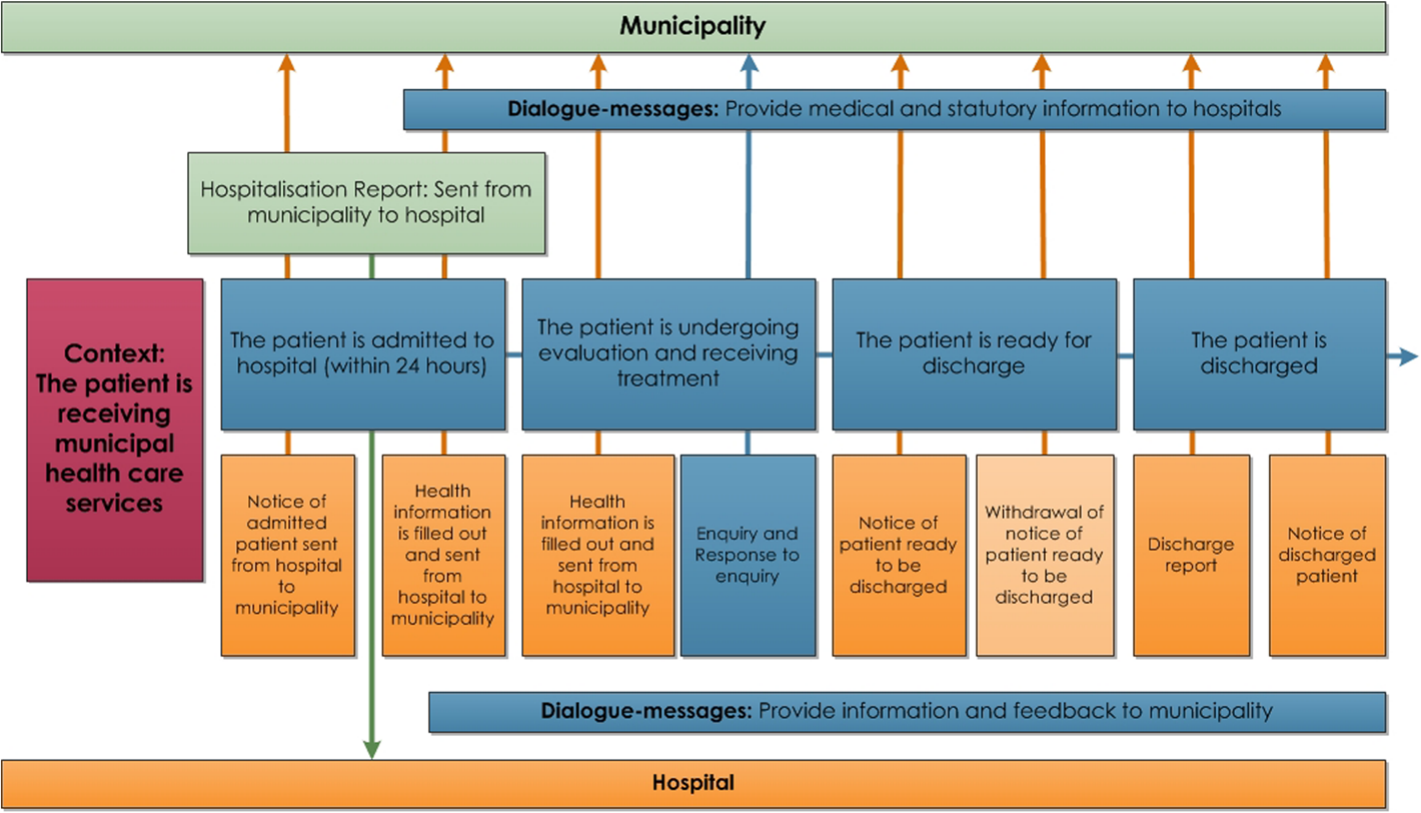
Blueprint of the PLO communication system [78]

The previous communication solution – using a fax for messaging between hospitals and municipalities, was based on two main messages sent by the hospital. The first message informed the municipality about the patient’s admission to the hospital/ward and provided information about the patient’s illness and expected date of discharge. The second message was sent by the hospital to the municipality when the patient was ready to be discharged. Care personnel in each ward were required to enter these “schemas” on their computer, print them out, and send them anonymously via fax to the relevant municipality. A follow-up phone call was always required to verify that the fax had reached the recipient and to reveal the patient’s identity. In the new solution, which is part of the electronic patient journal, the messages developed are correlated by name, structure, and function that reflect the specific patient case they are meant to support and result in a set of eight different (PLO) messages (Fig. 1).

Figure 1 depicts the designed flow of communication between a hospital and municipalities. This is an interesting document because it shows that, despite the PLO system being introduced to improve communication between hospitals and municipalities, the flow of communication was predominantly designed in a logic ‘from hospital to municipalities’ according to the direction of the arrows.

When asked how the system works in practice, respondent R5 in Ward C at the Hospital described the communication flow between the Hospital and the municipalities as based on the following main messages/steps:

1. A hospital nurse sends message 1, the notice of the admitted patient, to the ordering office (*Bestillerkontoret*) at the relevant municipality responsible for deciding the type and level of care the patient will need after hospitalization.
2. A nurse sends message 2 to the ordering office. This message contains health information about the patient plus expected discharge date. The tentative discharge date is decided after consultations among and between the nurses and doctor in charge of that patient and is usually communicated within 30 min after the decision has been taken.
3. The nurse sends message 3 to the ordering office when the patient is ready for discharge. This message normally describes the illness, treatment, and conditions (physical and mental) but also tends to suggest follow-up care for patients.
4. Through the ‘dialogue’ type of message, the ordering office usually communicates to the hospital the decision on care the patient will receive after hospitalization, and the hospital can comment/disagree with the decision and start sending messages back and forth, but it is the ordering office that has the last word.
5. Message 4 is the discharge protocol, which is a report to the municipality’s nurses and doctors who will be involved in the patient’s care after discharge.

Such description raises two key issues. First, we note that the Hospital ‘suggests’ to municipalities the follow-up care the patient should receive after discharge, although the ordering office has the formal authority to decide the type and level of care the patient needs. Second, we note that this ‘suggestion’ is more like a ‘strong recommendation’ when the ‘dialogue’ function has to be used to debate the issue.

In general, users of the new solutions have responded in a positive way to the new system. PLO has been revealed to be effective in overcoming the shortcomings of the previous solution. Although, according to a respondent in the Hospital, the use of free text space is a barrier to its full exploitation (R3), the new system makes the communication process smoother and more efficient (R18). The new solution enables health care workers to avoid the frustration of repeated calling when they get no answers (R19, R24) and affords them the possibility of answering in a calm moment without the interruption of other working activities (R24). Electronic messages did not completely substitute phone calls and are used in a flexible way. When information to be exchanged is ‘delicate and has nothing to do with the patient journal,’ then a phone call is the privileged means, and the content of the call would not be recorded in the system (R15). But, if there is something in oral communication that needs to be recorded, then users write a wrap-up message just after the dialogue to be documented in the electronic patient journal (R24). Written messages are also perceived as important in relation to eventual disputes concerning the payment regulation:

‘If a “notice of discharged patient” comes to us [ordering office] after 2.30 in the afternoon we do not have to pay anything… but they [the Hospital] can tell us later that they sent this message… and we can say “where is it?” We can show them there is no message about this patient going home and we avoid incurring a fine’ (R26).

Interestingly, our respondents stress what they perceive as misuse of the new system by their counterpart. From one side, hospital practitioners find that municipalities may be messaging too frequently. According to the respondents in the Wards B and C, hospital nurses have limited time available to handle PLO, compared with personnel at the municipal ordering office, who spend most of their time on the computer doing administrative work (R5). On the other side, municipalities also lament malpractices by the Hospital in using the messaging system. In particular, they reproach to the Hospital for not respecting the timing prescribed for sending a notice of discharge to the ordering office. It happens that the Hospital sends a message about a care service cancellation too late for the ordering office to re-route it to the home care unit (R13). Hospital sometimes forget to send the message of an admitted patient, and so they then send both messages – of a patient’s admission and discharge – at the same time (R15). This causes planning troubles for the municipality, which then has to arrange the care service in a constrained timeframe possibly leading to patients lying in hospital for longer than the hospital had planned (R24).

### Decision’s ownership

A key issue that emerged during our interviews concerns the hospital interference in the ordering office’s decisions. In the Norwegian health care system, the municipality, usually represented by a care professional sitting at the ordering office, is responsible for making decisions about the care service a patient receives after hospitalization. The ordering office is a unit that was introduced around 2000, following the new public management turn (R21) and the introduction of the purchaser-provider model, according to which there should be a separation between who approves a contract for care and who provides that service [58].

There are clear rules regulating this decision-making process: the hospital is responsible for describing patients’ conditions, including their functionalities, and, based on this information, ordering offices do their assessment and decide on the service to be provided. Despite the rules, this practice often generates controversies that unexpectedly are voiced in the ‘dialogue function’ included in the new communication system. According to our respondent in the Department A at the Hospital:

‘hospital practitioners sometimes pretend to know about the care services patients need after discharge and make explicit suggestions about them, although this is not their responsibility’ (R2).

A respondent at *Mal* Municipality explained this matter in terms of power dynamics:

‘We feel the balance is a little uneven…the hospital feels it has more power. The reform has given the municipality more power, but they don’t see it as such…they feel much more work pressure – not power … and when the hospital asks or tells what service the patient should have, the municipality feels it is being pushed and told what to do, without an opportunity to choose…’ (R12).

It is not rare that in difficult cases – when care personnel at the hospital and those at the municipality disagree about a decision to be taken – someone from the municipality personally visits the patient (R11) because sometimes ‘what the municipality observe and what the hospital observes is different’ (R26). Very often, the disagreement is around the kind of care, because, as one of our respondent explained, the hospital tends to suggest a more intensive level of care than the municipality (R16). Decisions become even more complicated to make when hospital practitioners ‘promise’ (R20) a certain care service to the patient or his/her relatives:

‘If the doctors or nurses at the hospital have said that the right level of care is an institution [nursing home or similar], then it becomes really difficult for us to argue for something different… so, sometimes we have to take them to an institution [nursing home or similar] maybe for some days/a week and then home … we have to find a compromise’ (R22)

Although the ordering office has the formal authority to decide about the care of the patient, it is dependent on the hospital’s decisions, first regarding the expected date of discharge and, second, at the moment of actual discharge. It is worth noting there have been very few cases in which respondents have emphasized the need to cooperate – interact more intensively between municipalities and hospitals. Respondent R6 in the Ward D is among the few recognizing the importance of collaboration between the hospital and municipality for the discharge to be successful. R6 describes this hospital–municipality interdependence as follows:

‘…the problem is that for many patients we have a training responsibility [e.g., for home care nurses], and although we could do the training, there is no one to train. The question is then: should we start communicating discharge before training anyone? We cannot discharge a patient until the personnel are trained.’

What makes the discharge process challenging is also the fact that, at the hospital, the decision to discharge is taken and then changed several times, thus making it sometimes difficult for the municipality in planning for the solutions (R18, R24). Still, a respondent in the home care unit (R14) at *Mal Municipality* said the new communication system helps in monitoring the situation; the coordinators are used to reading the exchange of messages between the ordering office and the Hospital. A coordinator explained that it is also a matter of how serious the patient’s conditions are:

‘If I see that “difficult” patients are being admitted to hospital, I pay attention to the messages. But, if I see “easy” patients, I do not really pay attention to what is happening’ (R14).

Our interviews further revealed that the doctor–nurse relationship at the hospital plays a major role in the stability/instability of the decisions regarding discharge. Even though the doctor has the formal responsibility for that decision, the discharge decisions often tend to be nurse-driven, and nurses are trying to keep doctors aware of the need to respect fixed dates for discharge. The following quote is an example of the dynamics related to discharge decisions in one of the Hospital wards:

‘…when there are a lot of decisions and re-decisions, a lot of messages back and forth is irritating…[for the ordering office]. This often happens when nurses and doctors do not speak to each other earlier [during patients’ hospitalization]. When the team is working well, we [hospital staff] are much more precise in our information, in our suggestion about expected date of discharge. [A good team] is when nurses and doctors find it important to have a plan and remember to take decisions…sometimes the nurse is too quick to give information about discharge [to the municipalities], while the doctor needs more time to evaluate and treat the patient…sometimes there is a difference [of opinion] between nurses and doctors about what is realistic [in terms of expected date of discharge] … we [nurses] observe the patient from the time he comes to hospital to when he leaves … we see more or less everything that needs to be done … doctors are more focused on the specific illness…’ (R5).

### Different ‘languages’

According to one of the respondents of the Department A at Hospital, the challenges during the discharge process revolve partly around the differences in assessing patients’ need:

‘municipal health care providers and the Hospital do not speak the same language…The hospital focuses on medicine; the municipalities focus on function and rehabilitation…that is whether the patients can live alone, get dressed, use stairs etc.… This has always been a problem, but the implementation of PLO messages has shed light on this’ (R1).

It was somehow unexpected to discover that the design of the new ICT system does not incorporate a specific function for exchanging information about the patients’ activities of daily living (ADL). As a consequence, professionals currently tend to use the ‘dialogue function’ (and eventually phone calls) to exchange this type of information (R26).

Respondents on the municipalities’ side point out that there is a great variation in the quality of information transmitted by the Hospital on ADL (R19) but confirm that when the Hospital is asked about further information, it generally replies with satisfactory information (R26, R23). To overcome the problem of lack of information lamented by municipalities, a creative solution was developed by a nurse of Ward C in collaboration with some municipality colleagues (R4). This consists of a laminated pocket card to be used as a guideline in the department. When sending an electronic message to the municipality, the nurses would easily extract the card from their pocket and check whether they have filled the message form with the needed information. In this way, less dialogue messages would be sent back and forth on lacking information (R24, R4).

With the introduction of the new incentive scheme, decisions made on discharge are not only a question of principle but also have tangible economic consequences. Indeed, a reason why the hospital often negotiates with the ordering office about decisions is related to the payment regulation reform (*Betalingsforskrift*).

### Change in economic incentives

The payment regulation reform established that municipalities must pay a fee to the hospital for every 24-h delay in receiving a patient when he/she is ready to leave the hospital. Prior to the coordination reform, the municipality had 10 days to decide and plan the appropriate care service before incurring a fee. The hospitals, however, were not obliged to issue a bill for the fee. Currently, the rule has changed, and the payment regulation reform makes the fee mandatory. However, due to the poor data input and the outcomes of some legal disputes, we found that the Hospital actually issues bills for 60% of the extra days of recovery (R7). Regarding this loss, the representative of the Regional Health Authority explains:

‘the Hospital has really bad documentation on when the patient is reported “ready to be discharged” and if the patient is not reported to be ready for discharge when he/she is actually is, then the Hospital loses these days [meaning losing bills related to these days]…They [at the hospital] do not know exactly how they shall report this: instead of adding [in the accounting system] the extra days after discharge, they often delete the whole thing and start a new discharge period…this is something that is not going exactly as planned’ (R27).

The municipality, which does not want to incur a penalty, is forced to plan in advance. The experience and opinions of our interviewees vary substantially on whether the payment regulation has achieved the intended results. First, from the Hospital’s point of view, the new incentive system does not change the budgeting and income structure, as the income imputable to the new payment regulation and to the co-financing on hospital admissions on selected somatic health services represent a minor part of their income. It seems the Hospital issues the bills because it is ‘obliged’ to do so and may not find the benefits of the additional administrative duties and costs. The idea with this billing practice was that municipalities would have been encouraged to receive patients who need municipal services as fast as possible. The co-financing instrument to reduce the number of admissions to the hospital did not produce the anticipated effects because, as two of our respondents evidenced, municipalities have no responsibility in relation to sending patients to hospital (R22, R23). This co-financing instrument was, in fact, removed in 2015.

The question remains open concerning the fee fixed to 4,500 Nok per day: Has it really been set in a way that constitutes an incentive for municipalities? If one should judge based on the indicative costs of services provided at the municipality – around 8,000-9,000 Nok per day for “regular” care services and up to 50,000 Nok for the most advanced treatment (R27) – the answer would probably be negative. According to the respondent of the Regional Health Authorities (R27) in Norway from 2012 to 2014, the total number of bed days in hospitals decreased (−1.8%) in the region of competence, but the number of days in the Hospital after discharge increased (+3,000 patient days for the Hospital and + 20,000 patient days for the entire region). When asked about possible reasons for this increase, our respondent wondered whether ‘it is due to increasing readmissions, and then one can ask if it is the competences out in the field [primary care] that are poor or if it is the hospital that sends patients home too early’ (R27).

### Adapting to the new incentive scheme

Returning to the effects of the penalties, we collected divergent, sometimes contradictory views. One of the respondents (from ordering office) was determined to declare that he/she did not care about the payment issue when he/she made decisions, as he/she would consider only what was best for the patient (R23). One respondent (at Ward C) suggested that, when there is no free capacity, the ordering office, unable to offer nursing home care to a patient ready to leave the Hospital , can decide to redirect the patient to another service unit, such as the home care unit, in order to avoid incurring payment of a fee. When the Hospital suspects this is the case, it can raise the issue with the ordering office. In a different ward (Ward E), Respondent R9 has a different perception of this issue. R9 explained that municipalities tend to leave the patient at the Hospital longer than necessary because when care services at municipalities are fully booked, it costs less to pay the fee to the Hospital than to pay for services provided by other institutions. The respondent also explained that this practice is risky:

‘patients who do not need treatment in hospital should leave as soon as possible to avoid the risk of incurring unexpected infections’ (R9).

On the municipality side, we have collected different reactions regarding the payment issue. Respondents have stressed the importance of not incurring delays in taking care of patients after discharge and, thus, to pay the penalties. Other respondents have explained to us that patients may have to stay in the Hospital after discharge because they are assessed to be ready to be discharged too early (and much earlier than in the past). As a result, municipalities ‘are receiving patients who are sicker and sicker’ to the extent that sometimes the municipalities do not have ‘the competences to treat the patient in such condition at all’ (R17). Some of the interviewees have even suggested readmissions should be checked because they believe that with the coordination reform – and the focus on efficiency – length of stay in hospital before discharge is decreasing but against an increase in readmissions (R15, R26).

A solution some municipalities adopt to address the problem of receiving sicker patients than in the past is allocating some ‘observation-beds’ for patients who have been discharged but the ordering office is unsure (or not ready) about the service to be offered (R24). Such ‘observation beds’ have not been implemented in all municipalities. *Mil* and *Mol* Municipality adopted a different practice to address the unexpected discharge of patients requiring complex treatment:

‘we rather prefer to pay for extra days at the hospital instead of being unsure of whether we can take care of the patient or not’ (R21).

‘we have discussed the possibility of having these “three-day observation beds” but concluded that we do not need them; we would rather give the patients a short-term place in a nursing home for a week and then we see…’ (R23).

In relation to the use of observation beds one of the respondent told us that a similar solution was experimented in 2009 (and closed down in 2013), in which an ‘intermediate unit’ was developed at the Hospital, in collaboration with four related municipalities, to manage the transition of patients from hospital to municipal care (R8).

Besides introduction of ‘observation beds,’ municipalities, to cope with undergoing changes, had to upgrade the competences of their personnel (R25) by receiving training from the Hospital but also hiring more specialized staff, as exemplified in the following quotes:

‘there is a team of three nurses at hospital who go out and train our [municipalities] nurses on special procedures we did not do before…at the nursing home they have hired new personnel so there are more nurses during the same shift to deal with the new procedures and the complexity of the patients’ (R23).

‘before, we had various supervisory doctors who had part-time positions; now, we have a supervisory doctor in a permanent position, and doctors visit patients at short-term three days per week, while before, they did visits one day per week’ (R24).

In some cases, the need for special procedures has also led municipalities to rely on private health care institutions:

‘we struggle a lot to have competent people for very special cases, so I think we are getting a new market here for private companies for very difficult cases coming to nursing homes and to help institutions, and I do not think this was the intent of the coordination reform’ (R21).

The fact that the economic considerations (the penalty in particular) might interfere with decisions on care services to be offered has been raised as a problematic consequence of the reform; the feeling is that it may be money that prevails, not the patient (R2).

## Discussion

The effects of the coordination reform and the two top-down instruments the Norwegian health authority promotes – ICT-based communication and economic incentives – have clearly transcended the aims ascribed to these. The two instruments interact with each other and with the broader context of arrangements and regulations in which they have been ‘implemented.’ A consistent overall picture of the effects of the two measures is difficult to draw because of the ‘in becoming’ nature of the processes explored and the variety of organizations and individuals involved. There are at least three issues that are worth discussing, as they might guide future policies directed to improve care coordination.

### Information needs and communication

Similar to studies on the use of electronic communication between nurses in home care and general practitioners [59, 60], we found that the users of the new PLO messages recognized the benefits of the new ICT system for exchanging information and communicating. In particular, the new system seems to fit well with the nature of work in both settings. Users at the Hospital perceive as one of the major benefits the possibility of sending and collecting necessary information without the pressure of being reached ‘on’ the phone. Users at municipalities feel more secure in having a written track of all the communication back and forth from the Hospital on which they can also rely in case of disputes. This points to the accountability role of the new technology [27].

However, if we flip the coin, both benefits reflect some reciprocal dissatisfaction. Professionals at the Hospital seem to be relieved by the decreasing need of synchronous dialogues with their colleagues at Municipalities. They reveal a general tendency of considering communication one way, where the hospital is the sender and the municipality the receiver. On the other side, municipality care personnel signal a certain mistrust on the Hospital’s ability/willingness to provide them with appropriate and timely information and see the new system as a tool for documenting shortcomings in the Hospital’s communication to municipalities. Documentation of the dialogue in the electronic patient journal also means a more formal dialogue, with the clear benefit of more extensive and easier exchange of information, while possibly reducing valuable informal conversational evaluations. It is evident from our study that the system’s blueprint which articulates the steps and timing of communication between the two actors does not automatically translate into a smoother and more timely flow of information exchange.

When asked about how the collaboration with the counterpart works, our respondents focused on the discharge process. In the new ICT system, there is no standard for the exchange of information on patients’ ADL that is fundamental to the ordering office at the municipality for its main task to assess patient’s conditions and decide after-hospitalization services. There are several reasons for the absence of a space to accommodate this information in the current system. First, as for all new ICT technologies, PLO was introduced while upgrades of the system were in progress. This means that this function might be included in the future. Second, translating ADL information in a standard format, likely based on a numerical scale, poses several challenges, not the least in terms of finding a common understanding of ADL and of the risks associated with a non-continuous (or erroneous) update of this critical information. Furthermore, our study has identified a further challenge. Similar to a previous study illustrating the cultural diversity between hospital and community nurses [40], we found that community professionals are often not satisfied with assessments provided by the Hospital because their expertise in assessing patients’ conditions is different from that of the Hospital. As a consequence, the assessment of a patient’s condition can follow different paths. Typical is a scaling up from the use of the ‘dialogue function’ to ask additional information when the hospital is lacking in the information provided to municipalities, to phone calls when disagreements among the parties do not allow managing the conversation by messaging, to direct visits to patients, usually when a resolution to the tension has not been found. This pattern is in line with what has been highlighted in a previous study that ‘IT can support the transfer of data in the post discharge period but coordinated decision making requires dialogue and agreement among different providers and between providers and patients’ [2].

Although tensions between the two health care actors during the discharge process are not an exception, cases where the collaboration runs smoothly are common. Collaboration appears to run smoothly when professionals at the Hospital take seriously the information needs of their colleagues at the primary care level. The pocket card created by a hospital nurse to be used as a reminder on which information (on ADL) to send to municipalities is an episode that shows a certain sensibility and respect for the work of colleagues on the other side [61]. Electronic messages between hospital and municipalities made the Hospital more aware of its duties during the discharge planning process as was expected to happen before the introduction of electronic patient journal in hospitals [62].

### Polarizing economic incentives

The punishing incentive scheme introduced by the coordination reform appears to collide with other policies which push hospitals to increase productivity and slow spending growth [63]. Our case clearly shows that, while the Hospital has adapted to the demands for efficiency by shortening the length of stay at the Hospital, municipalities seem to adapt more gradually (slowly) to earlier discharge. The scenario that an earlier discharge process opened up at primary level of care is varied (possibly confusing), depending on how the sanctioning incentive has been perceived by each municipality and on the capacity of municipalities to upgrade the existing competences and services. Essentially, patients in need of after-hospitalization care who cannot be received at municipality have to remain at the Hospital longer than necessary or can be transferred into ‘observation beds,’ which entails adding a further step in the patient pathway. A punishing incentive scheme, such as a penalty for delays in receiving patients ready for discharge, without a systematic strategy for developing the right arrangements to accommodate their needs, can put patients’ safety at risk and is in contrast with the ambition of advancing care coordination. The reform has generated a series of chain effects that have ‘forced’ local authorities to organize, more or less temporarily, new care services such as observation beds or cooperation with private actors for service provision [64].

We found that most animated conversations between hospitals and primary care are triggered by disagreements about the moment of discharge and the underlying economic incentives. This creates a somewhat paradoxical situation. On the one hand, the coordination reform aims at improving coordination between primary and secondary care, and the PLO communication system is a platform for interaction intended to improve communication and, thus, facilitate collaboration. At the same time, the payment regulation introduced by the coordination reform brings about a ‘win–lose’ logic that, rather than connecting and integrating the two organizations, pegs these against each other. Following the economic incentives, the organizations involved tend to prioritize their own, rather than the joint interests.

This ‘win–lose’ logic clearly nourishes the asymmetric pattern of collaboration between hospital and municipality [19]. The fact that a punishing incentive has been addressed to the municipalities, but not to the Hospital – for instance, when they delay in sending the notice of discharge – strengthens once more the privileged position of hospitals. Our study confirms that there are “no equal voices of participants” [47] and that the large hospitals sometimes impose “a ‘hospital-centred’ view of healthcare – particularly with regard to providers who care for patients post-discharge” [9]. A clear example is when the Hospital ‘strongly recommends’ to municipalities which care service the patients should receive after discharge, or when the Hospital communicates to the patient and/or family members which after-hospitalization service he/she will receive. Doing so, the Hospital attempts to expand their jurisdiction over municipalities’ tasks [5]. Sharpening the distinctions between the parties’ roles appears to lead to tougher negotiations and stronger efforts to maintain individual privileges and responsibilities, rather than to engage in open dialogue about patients. In particular, municipalities perceive the workload of changes in place mostly on their charge and do not see this to be counterbalanced by any empowerment implied by the reform. This raises the question whether those to whom power was devolved were equipped or minded enough to engineer the shifts [43]. However, to avoid further polarization of interests and contrasting logics, a shift is needed toward financial incentives more “consistent with an integrated health care system” [65].

### The need for shared vision and acting jointly

We found that legal or regulatory requirements play an important role in relationships between hospital and municipalities [66]. The reform has reaffirmed the distinction of roles and responsibility between the two levels of care, thus reinforcing their distinct jurisdictional domains [67]. The challenge is to find a way to shift from collaboration based on local economic incentives to collaboration based on the idea of joint responsibility. Such a shift is likely to involve creating shared incentives [68] and shared performance measures [69] so that both hospitals and primary care physicians have incentives to offer post-discharge care needed for a smooth transition between a hospital and home. In a context such as the one this paper examines, health policymakers need to address a fundamental question: Should decisions about health care services after discharge continue to be an exclusive matter of the care professional at the municipality level or should a joint decision-making across health institutions be favored to improve the coordination process and its efficacy also in terms of patient care? Furthermore, if the intention is to shift toward a more patient-centred approach [70], decisions regarding after-hospitalization services should include the voice of patients more systematically.

Effective coordination between primary and hospital care depends on the quality of relationships among different professionals [62]. Therefore, improving coordination requires the development of cooperative voluntary inter-organizational relationships. Complex and tacit knowledge, as the one needed in health care settings, is more readily connected and accessed through strong relationships [9]. Accordingly, more attention has to be devoted to the design of mechanisms to manage inter-organizational relationships. In a context where ‘centrally orchestrated coordination’ [71] is ruled out, developing a tightly coupled health care system has to follow other paths. These are likely to involve making the project a joint endeavor [72], emphasizing common understanding instead of accountability, and transforming outsiders into insiders by engaging in conversation about operational aspects or even by designing ‘boundary-crossing roles’ [73]. To overcome the hurdle of the preconceptions of other health care providers, recognizing and understanding each other’s work practices, or ‘interconnected practices’ [74], is crucial. This requires better mutual understanding and necessitates a social perspective-taking aptitude as “the ability to understand how a situation appears to another person…to put oneself in the place of others and recognize that other individuals may have points of view different from one’s own” [75]. Superior perspective taking has been linked to positive outcomes that include more effective cooperation [75], better communication [76], and the resolution of conflicts [77], all of which appear crucial in care provision managed and practiced as joint collective responsibility and are conditions for the entire health care network to co-develop.

Our study confirms that enhancing care coordination is a “multifaceted phenomenon and more complicated than health policy suggests” [11]. Improving coordination in complex settings, such as the health care system, requires complex instruments that need to be monitored for the effects they produce independently of the aim declared and ascribed to them [44].

Despite the potential contribution of the current study, there are some limitations worth noting. First, the use of a convenience sample of a care professional derived from a limited number of divisions at the Hospital, as well as a subset of the 21 municipalities in the catchment area of the Hospital, limit the generalizability of the findings. Future research may use a more diverse sample by obtaining data from a larger number of professionals at the Hospital as well as at municipality level.

## Conclusion

This study has examined the effects of two government measures intended to improve coordination among healthcare organizations. The study sheds light on the controversial nature of top-down instruments, especially when these imply spanning across professions and organizational boundaries. The challenge in coordinating primary and secondary care evidenced in this study is to find a way to shift from collaboration based on local economic incentives to collaboration based on the idea of joint responsibility and voluntary cooperative relationships which requires ‘common and joint’ incentive schemes. Our study adds to previous studies on care coordination by directing attention to the need to harmonize and integrate policies and underlying incentive schemes across organizations within the health care system. We believe that this exploratory study offers some guidance to policy makers especially when evaluating incentive schemes, and it informs health professionals at primary and secondary care level on the reciprocal communicative shortcomings that affect coordination across organizations. We also hope our study can spur health service researchers to further develop our understanding of the mechanisms through which coordination between primary and secondary care can be achieved.

## Abbreviations

ABF: Activity-Based Funding
ACOs: Accountable Care Organizations
ADL: Activities of Daily Living
EHR: Electronic Health Records
ICT: Information and Communication Technology
PLO: Pleie- og omsorgsmeldinger
REK: Regional Committees for Medical and Health Research Ethics
